# Developing Content for a mHealth Intervention to Promote Postpartum Retention in Prevention of Mother-To-Child HIV Transmission Programs and Early Infant Diagnosis of HIV: A Qualitative Study

**DOI:** 10.1371/journal.pone.0106383

**Published:** 2014-09-02

**Authors:** Thomas A. Odeny, Maya Newman, Elizabeth A. Bukusi, R. Scott McClelland, Craig R. Cohen, Carol S. Camlin

**Affiliations:** 1 Center for Microbiology Research, Kenya Medical Research Institute, Nairobi, Kenya; 2 Department of Epidemiology, University of Washington, Seattle, Washington, United States of America; 3 School of Medicine, University of Washington, Seattle, Washington, United States of America; 4 Department of Global Health, University of Washington, Seattle, Washington, United States of America; 5 Department of Obstetrics and Gynecology, University of Washington, Seattle, Washington, United States of America; 6 Department of Medicine, University of Washington, Seattle, Washington, United States of America; 7 Institute of Tropical and Infectious Diseases, University of Nairobi, Nairobi, Kenya; 8 Department of Obstetrics, Gynecology and Reproductive Sciences, University of California San Francisco, San Francisco, California, United States of America; 9 Center for AIDS Prevention Studies, University of California San Francisco, San Francisco, California, United States of America; UCL Institute of Child Health, University College London, United Kingdom

## Abstract

**Background:**

Maternal attendance at postnatal clinic visits and timely diagnosis of infant HIV infection are important steps for prevention of mother-to-child transmission (PMTCT) of HIV. We aimed to use theory-informed methods to develop text messages targeted at facilitating these steps.

**Methods:**

We conducted five focus group discussions with health workers and women attending antenatal, postnatal, and PMTCT clinics to explore aspects of women's engagement in postnatal HIV care and infant testing. Discussion topics were informed by constructs of the Health Belief Model (HBM) and prior empirical research. Qualitative data were coded and analyzed according to the construct of the HBM to which they related. Themes were extracted and used to draft intervention messages. We carried out two stages of further messaging development: messages were presented in a follow-up focus group in order to develop optimal phrasing in local languages. We then further refined the messages, pretested them in individual cognitive interviews with selected health workers, and finalized the messages for the intervention.

**Results:**

Findings indicated that brief, personalized, caring, polite, encouraging, and educational text messages would facilitate women bringing their children to clinic after delivery, suggesting that text messages may serve as an important “cue to action.” Participants emphasized that messages should not mention HIV due to fear of HIV testing and disclosure. Participants also noted that text messages could capitalize on women's motivation to attend clinic for childhood immunizations.

**Conclusions:**

Applying a multi-stage content development approach to crafting text messages – informed by behavioral theory – resulted in message content that was consistent across different focus groups. This approach could help answer “why” and “how” text messaging may be a useful tool to support maternal and child health. We are evaluating the effect of these messages on improving postpartum PMTCT retention and infant HIV testing in a randomized trial.

## Introduction

Early diagnosis of HIV among infants born to HIV-positive mothers is a critical component of prevention of mother-to-child transmission of HIV (PMTCT) [1]. The World Health Organization (WHO) recommends that infants of HIV-infected women undergo HIV testing at six weeks. WHO estimates that in low- and middle-income countries in 2010, only 28% of HIV-exposed infants were tested for HIV within two months after birth [2]. Similarly, a high proportion of HIV-positive pregnant women are not retained in PMTCT programs after delivery [1], [3]–[5]. One potential solution for improving maternal retention in PMTCT programs and increasing the rates of early infant HIV diagnosis is the use of mobile phone technology for health (mHealth). We have previously used text messaging to improve clinic return rates for other HIV prevention interventions [6]. The United Nations Children's Fund encourages PMTCT programs to utilize mobile phones in health interventions because of the high levels of access among enrolled mothers [7]. Mobile phones have successfully been used to support other aspects of HIV care and treatment. For example, two recent randomized controlled trials found that mobile phone text message interventions significantly improve adherence to antiretroviral therapy [8]–[10]. Despite strong evidence of efficacy, to our knowledge the theoretical foundations for these interventions have not been reported in the literature. This makes it difficult to interpret *why* the interventions were efficacious [11], and *how* they can best be transferred to other regions. Further, the authors of both landmark trials concede that mHealth interventions that affect behavior change in one context might not work in another [12]. SMS interventions, like other health behavioral interventions, need a theoretical framework to explain how each aspect of the intervention relates to each construct of the theory; in other words, a causal framework illustrating why a particular intervention is hypothesized to be successful [13], [14]. If the theory can explain the *why*, then it might be possible to apply and adapt the framework to other health behaviors in other populations [11], [15], [16]. Despite a rich foundation in theory that has been developed to understand HIV-related sexual risk behavior change interventions [17], the application of behavioral theory to SMS intervention studies for HIV is nascent. Further, the qualitative process of developing SMS interventions in the HIV field is rarely documented. We address this gap by describing our process of developing a theory-informed SMS intervention. Our aim was to develop a set of individually tailored, culturally appropriate text messages targeted at improving uptake of infant HIV testing rates and maternal retention in PMTCT programs.

## Methods

### Setting

This study was conducted in the Nyanza region of Kenya, an area with high adult HIV prevalence in general (14%), and among pregnant women in particular (18%) [18], [19]. Study participants were recruited from among patients and providers at health facilities supported by Family AIDS Care and Education Services, a President's Emergency Plan for AIDS Relief /Centers for Disease Control funded program which is implemented as a collaboration between the Kenya Medical Research Institute and and the University of California San Francisco [20]. These health facilities all offer PMTCT services, including testing for infant HIV infection.

### Sampling design and participants

Purposive sampling was used to recruit participants for five focus group discussions (FGD). These groups were selected to represent the population of women who would be potential recipients of the text messages, and health workers who would interact with them routinely. General announcements were made asking women to take part in discussions to help design culturally appropriate, educational text messages for improving early infant diagnosis of HIV. Health workers at maternal and child health (MCH) clinics were approached in person by a member of the study team and invited to participate. Between eight and ten participants each were recruited for four different focus groups: health workers from maternal and child health clinics; women attending antenatal clinics; women attending postnatal clinics; women enrolled in PMTCT programs. Eight other women representing each of these four groups were recruited for a fifth follow up FGD to further develop, refine and pretest messages. We also purposively recruited four clinic staff members to participate in cognitive interviews for a final pretesting of messages. They were selected to represent both clinical and community health service providers with experience working in the study region.

### Data collection

After completing their clinic visit or clinic duties for health workers, participants were brought to a private room where trained moderators led the discussions in English, Kiswahili, and Dholuo, the primary languages spoken in this region. In addition to a moderator, each focus group had a note-taker documenting the discussion in detail. All participants gave oral consent to participate in the study and were then assigned identification numbers that were used in place of their real names. Participant demographic information was then collected on a separate form, distinguishing each woman by her assigned identification number. Participants received a transport reimbursement of KSh250 (approximately US$3) and refreshments during the discussion.

The content of the study's structured focus group discussion guide was informed by the Health Belief Model [13], [21], [22], as well as findings of prior empirical research on factors that may influence attendance at postpartum clinic appointments and infant testing. We referred to the Family Health International handbook on focus group research [23]. The moderator introduced the project and described how the participants' general experiences and opinions would be helpful to design the SMS intervention, then introduced a series of topics approximately organized according to the constructs of the HBM, but permitting additional topics to emerge in the discussion. The first topic, *perceived susceptibility*, explored participants' perceptions of the risk of their (or others') children being HIV positive, and the risks associated with failure to attend maternal and child health clinics for early infant testing. Questions for the second topic, *perceived severity*, elicited participants' perceptions of the consequences of failure to attend clinic after delivery, and the consequences of HIV testing for infants. The third topic, *perceived benefits*, explored participants' beliefs about the reasons why women should return to clinic and bring their children for HIV testing following delivery, and their perceptions of the potential positive benefits of doing so. We further probed about the kinds of things that may need to be done in order for women to understand those benefits. For the fourth area, *perceived barriers*, we sought to determine the material costs, assumed by the mother, of returning to clinic after delivery and bringing her child for HIV testing. We also asked women to describe what might prevent them from returning to the clinic for themselves or their infant. We then probed about the kinds of assistance that women would need in order to help them overcome these challenges, whether related to incentives or messages of reassurance. The final discussion topic addressed both *cues to action* and *self-efficacy* [24], [25]. To develop the best potential “cues to action”, we asked participants specific questions concerning the kinds of text messages that would potentially help women and their families understand more about the importance of postnatal clinic visits and HIV testing for their children, including their content, timing, and frequency. To explore aspects of “self-efficacy”, we asked participants what sorts of messages may best encourage women and elicit their self-confidence in their ability to attend postnatal clinic visits and bring their newborns in for HIV testing. Finally, before closing the discussion and thanking participants, the guide allowed participants to offer other ideas, besides sending messages, to help women return for post-natal clinic visits and bring their infants for HIV testing [13], [22], [26]. The focus group discussion questions are displayed in Table 1.

**Table 1:** Focus group discussion questions grouped by Health Belief Model constructs.

Perceived susceptibility
What do people think about women visiting clinics after giving birth?
What do people think about bringing children for HIV testing soon after birth?
In your community, who makes decisions about these things in a family? The husband? The wife? Or another family member?

Perceived severity
Why do some women fail to return to clinic after giving birth?
Why do you think some women fail to bring their children back to clinic for HIV testing after birth?
What are some of the consequences of failing to return to clinic after giving birth?
What are some of the consequences of failing to bring infants for HIV testing after birth?
What things can be done to help women return to the clinic after giving birth?
What things can be done to help women bring their infants for HIV testing?

Perceived benefits
What are some of the reasons why women should return to clinic after giving birth?
What are the potential positive results of returning to clinic after giving birth?
What are some of the reasons why women would want to bring infants for HIV testing after birth?
What are the potential positive results of bringing infants for HIV testing after birth?
What kinds of things may need to be done in order for women to understand the benefits of returning to clinic and bringing their infants for HIV testing?

Perceived barriers
What are some of the material costs related to returning to clinic after delivery and bringing infants for HIV testing? By material costs, I mean cost of transport, time taken away from work or other duties, and such like things, distance that you have to travel, and similar things.
What things would women worry about that would prevent them from returning to clinic, or prevent them from bringing their infants for HIV testing?
What kind of assistance would women need in order to help them overcome these challenges? Incentives? Reassurance?

Cues to action & Self-efficacy
If we were to send pregnant women SMS text messages, what are the kinds of messages that would help them and their families understand more about post-natal clinic visits and HIV testing for their infants? When should these messages be sent? What time in the post-partum period? What time of day? How frequently?
What kinds of messages would help them understand the importance of post-natal clinic visits and HIV testing for their infants?
What kinds of messages would help them understand the consequences of failure to attend clinic and/or bringing their infants for HIV testing? When should these messages be sent? What time in the post-partum period? What time of day? How frequently?
What kinds of messages do you think women may want to receive to help them better understand the potential benefits of returning to clinic and bringing their infants for HIV testing? When should these messages be sent? What time in the post-partum period? What time of day? How frequently?
Are there specific messages that you think women should receive to help them overcome material and psychological challenges that prevent them from either returning to clinic after delivery or bringing their infants for HIV testing? When should these messages be sent? What time in the post-partum period? What time of day? How frequently?
Apart from just sending messages, what are the other things that can be done to help women return for post-natal clinic visits and bring their infants for HIV testing?

### Data analysis, message development, and pretesting

Audio recordings from the FGDs were transcribed and then translated into English. The data were first coded and analyzed according to the construct of the HBM to which they were related. From these broad themes, sub-themes emerged through the context of the discussion that were used to design a sequence of focused text messages. The first draft of proposed messages were developed from the major themes and subthemes by a team of content experts. The team comprised a behavioral scientist and two physician-epidemiologists with expertise in the fields of HIV prevention and treatment, and experience in text messaging interventions for HIV prevention. The draft interventional SMS messages were then pretested in a follow-up focus group with systematically selected participants from each of the four groups (health workers from maternal and child health clinics, women attending antenatal, postnatal, and PMTCT clinics) in order to develop the optimal wording and phrasing of the messages in the three languages. We used a cognitive interviewing approach [27] for two stages of pretesting of the messages. First, participants were shown written examples on an easel notepad of different SMS messages (in English, Kiswahili, and Dholuo) to be used in the third trimester of pregnancy and the postpartum period. Participants were asked to comment on their understanding of and feelings toward the messages, as well as how to improve the text. During the discussion, the moderator edited the messages on the easel notepad in order for all the participants to see the changes and agree on the most appropriate text for each of the messages in all three languages. Participants were invited to write on the easel pad if they wished to edit the messages themselves. Following the development of the messages in the focus groups, we carried out a second pretesting of the messages in individual cognitive interviews with selected volunteer clinicians and non-clinical program staff members. These individuals included an expert in community liaison, a nurse experienced in providing clinical and community HIV prevention services, a local social scientist, and a community mobilizer/community health worker. Each individual further refined the messages, ensured that the word count was within the limits for a typical SMS, and ascertained that the translations between the three languages retained the same meaning. Their suggestions were then reviewed by the initial team of content experts, resulting in a final set of messages. The review of messages by health workers was limited to ensuring that the word count for SMS was acceptable, and that intended meanings were not lost in translation to the local languages.

### Ethical review

This study was approved by the Ethical Review Committees of the Kenya Medical Research Institute, the Human Subjects Division at the University of Washington, and the University of California San Francisco Committee on Human Research. All participants provided oral informed consent. The ethical committees approved of this consent procedure. A waiver of the requirement to obtain written documentation of consent was allowed by the ethical review committees because the research presented no more than minimal risk of harm to subjects and involved no procedures for which written consent is normally required outside of the research context. In order to provide subjects with the required consent-related information about the research, we provided both an oral explanation of the research and a written information sheet. Specifically, the focus group moderator read out consent-related information to participants from an information sheet. Participants were informed that participation would be voluntary, discussions would be confidential, and that they could withdraw from the focus group at any time with no negative repercussions. The moderator then gave each participant a copy of the information sheet, which contained all consent-related information about the study including descriptions of the purpose of the focus groups, topics to be discussed, and expected risks and benefits. Participants were then individually asked for permission to take part in the discussion as part of the oral informed consent process.

## Results

### Participant characteristics

We conducted five focus group discussions, with a total of 41 participants. The median age was 28 years (inter-quartile range [IQR] 24–31), 34 (83%) were married, 39 (95%) were Christian, 27 (66%) were employed, and 24 (59%) had at least secondary-level education. The median number of living children reported was one (IQR 0–2). Participant characteristics for each focus group are summarized in Table 2.

**Table 2:** Participant characteristics.

	Health worker (n = 9)	Antenatal clinic attendees (n = 8)	Postnatal clinic attendees (n = 8)	*PMTCT clinic attendees (n = 8)	Mixed focus group (n = 8)	Overall (n = 41)
Characteristic	n (%)	n (%)	n (%)	n (%)	n (%)	n (%)
Age – median (inter-quartile range)	30 (29–40)	25 (21–28)	30 (28–36)	23 (21–25)	30 (28–36)	28 (24–31)
Married	4 (44)	7 (88)	8 (100)	7 (88)	8 (100)	34 (83)
Religion – Christian versus Other	9 (100)	8 (100)	7 (88)	8 (100)	7 (88)	39 (95)
Education – secondary or higher	9 (100)	4 (50)	3 (38)	5 (63)	3 (38)	24 (59)
Living children – median (inter-quartile range)	2 (0–2)	0 (0–2)	2 (1–4)	1 (1–2)	2 (1–4)	1 (1–2)

*PMTCT, Prevention of mother-to-child transmission of HIV.

### Perceived susceptibility to, and severity of HIV and other childhood illnesses

In this high HIV prevalence area of Kenya, women often perceive themselves and their infants to be highly susceptible to HIV and other illnesses:

*“When a mother fails to attend the clinics, she may be HIV positive yet she will just go and give birth then goes ahead to breastfeed this child. This is likely to cause HIV transmission to the child…” – 22 year old married participant (postnatal clinic)*

*“If we leave out the issue of HIV, there are lots of things that can harm the baby… if he is not immunized with polio vaccine or the others which we know, the child may grow up in poor health and at the end he may even become paralyzed …” – 35 year old married participant (PMTCT clinic)*

### Fears of returning to clinic, and perceived consequences of failure to return

#### Participants noted that infant illness was the main consequence of failure to return to clinic after delivery

Women were aware of the severity of HIV infection for their infants. However, fears of HIV and of disclosure keep many women from returning to clinic for postpartum visits and infant HIV testing. Many women associate the clinic with learning one's HIV status, and even death. Those who have yet to disclose their status to their partners fear they will have to reveal that they are positive if they go to the clinic for delivery or postpartum visits.

*“On many occasions I see people prefer mostly the traditional birth attendant…these ones do not ask you about your status… If I go back to hospital I will discover the baby's status and know mine again. Therefore, I would rather stay there to avoid much stress.” – 27 year old participant (antenatal clinic)*

Several HIV-positive women reported fearing that if they return to the clinic to have their child tested, they will learn that their infant is also HIV-positive. These women discussed how knowing that HIV infection tends to exact a more severe toll on children may make women feel anxious or psychologically disturbed. Participants were aware that HIV positive children who fail to return to clinic for HIV diagnosis are more likely to fall sick and require hospitalization than those who return to clinic and undergo early infant HIV diagnosis. This also leads to higher financial costs:

*“…the consequence of not going to the clinic is that you may require to use more money, you may even require to sell a cow if you had one, if the baby is in the ward for treatment. This implies that you ought to have gone to hospital to avoid using a lot of money…” – 28 year old participant (antenatal clinic)*

Most participants identified, in particular, lack of information, transport costs, religious beliefs, fear of HIV testing, and fear of disclosure of HIV status, as important barriers to postnatal clinic attendance. In addition, young mothers may be unaware of the importance of antenatal, delivery, and postpartum care, or may be dissuaded by other mothers who never took their children to a clinic.

Participants reported that some women might opt to visit traditional healers due to the cost and time necessary to travel to a clinic and to take time off of work. Participants felt that women worry about the long queues at the clinic and having to wait for hours to be seen by a clinician, often missing work or leaving other children at home. Many women live in remote areas and travel long distances to arrive at the clinic. Moreover, participants complained that the line to wait to be seen at the clinic can be six to eight hours.

*“These nurses … are not ready to serve you. When it is time for lunch they slip away … leaving you there waiting. Such kind of things can discourage one and make her become reluctant in going to the clinic because when you come you cannot get the appropriate services.” – 35 year old participant (PMTCT clinic)*

While lack of information, cost, travel time, and wait time impede many women from attending, participants noted that failure to go to the clinic may result in complications that require hospitalization which ultimately accrues a significantly higher cost for the family.

### Perceived benefits

**Benefits of returning to clinic after birth.** The majority of participants recognized infant and maternal wellness as the central positive outcomes of returning to clinic after giving birth. They identified immunization and HIV testing for infants, nutritional counseling and maternal health assessment as benefits of clinic return. Participants also noted that the clinic teaches HIV-positive women about family planning methods and how to exclusively breastfeed to minimize HIV transmission.

*“If you bring the baby to the clinic, you do so to check on his status. First of all you get information that when you deliver, you bring the baby to the clinic and you are taught how to take care of him, what to do if you have to breastfeed him even when you have to be away from home.…” – 30 year old participant (PMTCT clinic)*

*“Another fact that I think is making these mothers to come is family planning. I realize that a good number of them are starting to practice family planning methods.…” – 29 year old female nurse (health worker)*

Participants noted that in addition to detecting early childhood illness, if a child is identified as HIV-positive, then the clinic team can discuss and apply necessary medical interventions. If the child is found to be HIV-negative, then it is important to continue educating the mother and monitoring the child to prevent mother-to-child transmission.

Finally some women highlighted that a child is “innocent” and at the whims of his caregivers to provide the optimum care, and therefore should have the opportunity to be tested for HIV to enable early and preventative treatment.

*“It is never a good thing, especially for the innocent child— because for you, you may have known your status then decline to take the baby there to be tested so that when found positive, he may be put on care early enough, together with the appropriate treatment. That way, you make a mistake for the baby, because if he is infected, you will neglect his health just like that. And if he is not infected, maybe he has been born free of HIV but [the] breastfeeding and mixed feeding you do to him will compromise his health. This is not good— it may have a bad outcome.” – 36 year old participant (PMTCT clinic)*

### Perceived barriers: health systems and structural

Participants cited a range of practical, social, and psychological barriers many women face in attending clinic during the postpartum period. Participants identified poor communication between patients and healthcare workers as a barrier to getting a referral to a clinic closer to one's home. Participants explained that when nurses and clinical officers take the time to talk with and explain to women the specific reasons for coming back to the clinic, they often do. Yet, participants explained that the care in the clinic is often given without sympathy, and the medical personnel can be unfriendly and discouraging:

*“… we need friendly grounds. When I get to the clinic and realize I'm being treated unfairly, I may not come. You know we are also like babies, we also need people who care…. So the kind of clinic we may want to have are those that have friendly nurses to treat us well…. Let them understand each one of us because we are different.” – 27 year old participant (antenatal clinic)*

*“I think one issue that can make these mothers remember to come back to clinic, we have to be friendly to them.... ‘Cause if you talk to her rudely or you are cruel to her, she might not feel like coming back, so we have to be friendly.” – 43 year old female nurse (health worker)*

In addition to recommending that clinic staff should be more warm and compassionate to patients, participants offered numerous suggestions for how to help women overcome many of these material barriers that challenge women's ability to get to clinic. Participants suggested increasing the number of staff at the clinic to reduce wait times, building more health facilities to improve accessibility, and sending text message reminders.

### Providing cues to action and enhancing self-efficacy

The majority of participants identified education of pregnant women as one of the things that could be done to help women return to clinic after birth. Participants suggested that health workers should provide information and motivate women early in the pregnancy about the importance and reasons to return for infant testing, as well as the potential consequences of failing to return.

*“If they know the reason why they are coming then they will be coming. [If] the right information is given, a return date is given, that by this date you are supposed to come back, then they will be able to come.” – 24 year old female nurse (health worker)*

*“I think the mothers who do not want to bring their babies to the clinic should be given appropriate education on the benefits of taking the baby to the clinic, and what might happen if they do not take them.” – 32 year old participant (postnatal)*

Moreover, participants felt that health workers should emphasize the importance of involving and educating the partners in the decision to return to the clinic for infant testing to help remind and support the mothers to ensure the safety of the child.

*“Involving the partners …I used to see in another clinic, whereby when the mother comes for the first antenatal clinic visit they type some letters. Like every woman takes a letter to the husband, so that the next visit the husband accompanies the partner to the clinic.” – 40 year old female nurse (health worker)*

*“I feel it may be possible for a man to take responsibility of telling the woman to go to the clinic when the woman discloses her status to him so that he also joins in.” – 36 year old participant (PMTCT clinic)*

### Potential channels of information

Participants offered many suggestions regarding methods that can be used to provide important information for women, including phone reminders (text messages and phone calls) from the clinic.

*“If they have disclosed maybe you can text a message like maybe a reminder. Maybe she was supposed to come to the clinic today and by evening she's not come, so you are going through the files and you've realized she's not come. So you can send a message like, “Hi, you were supposed to report to the clinic today. Why didn't you make it?” – 31 year old female clinical officer (health worker)*

*“What could remind a mother who has delivered or an expectant mother to come back to the hospital is just to take the phone numbers of the patients. I mean we should avail them at the PMTCT office so that if you miss your appointment, it makes it easy for them to call and remind you” – 36 year old participant (PMTCT clinic)*

Some participants suggested that nurses communicate and work with traditional birth attendants to help urge women to go to the clinic after they have delivered. Several women suggested other forms of community involvement, such as through “mentor mothers” who can identify with the pregnant woman and inspire them through their own experience of taking their children to be tested, as well as through community gatherings of mothers (e.g., led by a chief or at a church) expressing the importance of going to the clinic after delivery.

*“The use of mentor mothers can go a long way because they are living examples. It is easier for someone to identify with someone who has the same problem as them. The mentor mothers are positive who have success stories of how they took care of their children and how the children turned out negative.” – 29 year old female nurse (health workers)*

*“The nurses can get in touch with the traditional birth attendants to invite the mothers who are not willing to go the clinic.” – 23 year old participant (postnatal clinic)*

### Participants' recommendations for messaging content

Table S1 contains a selection of quotes offered by focus group participants to support the inclusion of major themes into intervention text messages. In addition to exploring these topics, FGDs incorporated participants' specific suggestions for the content of text messages and schedule for their delivery in the intervention. Participants preferred messages that congratulated women on their delivery and those that had a personal touch. They suggested that text messages that were caring, polite, and encouraging would persuade women to return to clinic after giving birth. As one 27-year old woman mentioned, “First of all I may really want someone to congratulate me because this journey is really long, to reach the destination is no joke…I'll feel happy ‘cause we all need to be congratulated for the success.” Other comments included:

*“Everybody would wish to be loved…Meaning, the first word you use must sound loving… You may use words like ‘please’, ‘kindly’ and others like these. It could be one word that can generalize the others and after putting it that way, she will say for sure this person has talked to me politely and humbly.” – 35 year old participant (PMTCT clinic)*

*“…Being polite is very important… when you speak with people they associate hospitals—sadly, government hospitals – with people that are rude.... They need that touch; we need to be polite to the person who is coming. That will make the person come back.” – 30 year old male doctor (health worker)*

Several participants emphasized the importance of brief educational messages. One 20-year old woman said, “… it should be an educative message and should be precise, I mean very short,” and a 35-year old participant noted that, “You know SMS is a brief statement and you cannot make it too long. You just have to look for few words which can comfort someone or sound polite.”

According to most participants, potential messages should begin with a greeting, include the woman's name, congratulate her, ask if she has attended clinic, and give a brief educational message or appointment reminder, as illustrated in the following quotes:

*“You should be positive, you know; somehow, a positive message trying to convince them why it is important for them to come. Also important for someone to take a message very seriously: that personal touch. If you could put someone's name, like say she's called Mary. Use the name ‘Dear Mary’…” – 30 year old male doctor (health worker)*

*“You know if one calls you by name you know at least they recognize you and it is not like they sent the SMS for granted. So that would mean they know me and want me to go. In the first place, that would motivate me to go.” – 27 year old participant (antenatal clinic)*

There was consensus that messages should be sent in the daytime, preferably during regular work hours.

*“Definitely during the day.” – 40 year old nurse (health worker)*

*“…Office hours, eight or seven to six. I think that's going to be the best time, reason being that you know when you get a message and you don't know the number, people usually call back.” – 30 year old male doctor (health worker)*

*“Any time from eight to eight because at night people sleep early and so they do not even bother to read.” – 27 year old participant (antenatal clinic)*

Participants suggested that women may need to receive messages a few days before the appointment date to enable them to prepare. One 22-year old woman said, “Not immediately but when her appointment date is close by, maybe three days to, or one week to.” Other illustrative quotes were:

*“It should be sent a day before, like it should be sent today if you were to go tomorrow to enable you to prepare early.” – 24 year old participant (postnatal clinic)*

*“I would prefer to be reminded maybe three days in advance so that one may prepare herself even financially.” – 27 year old participant (antenatal clinic)*

Participants emphasized that text messages should not mention HIV or HIV testing as a reason for clinic return.

*“But you should not send one like this, ‘Please come; bring the baby for HIV test. ’ No!” – 22 year old participant (antenatal clinic)*

*“I believe that an SMS…can land anywhere…A message has no secret so if I tell you that you are HIV positive kindly come for testing, you know that one will demoralize me and I may faint, because that is my status which I wouldn't have wanted to share with another person.” – 27 year old participant (antenatal clinic)*

In addition to SMS messages, women also suggested using other media, such as educational posters at marketplaces and messages over radio and television, which would be sure to reach women without phones and those in rural areas. Moreover, some participants proposed working with community leaders, such as the chief or an elder to help remind people.

The research team utilized these findings to craft a set of text messages and a schedule for their delivery. The first draft of interventional SMS text messages was presented in follow-up FGDs with selected participants from each of the groupings in order to develop optimal phrasing in local languages. On the basis of findings of this first stage of pretesting, we then further refined the messages, and pretested them in individual cognitive interviews [27] with selected volunteer clinicians and non-clinical program staff members. After a final stage of analysis and refinement, the messages were finalized for the intervention. Table 3 contains the final set of text messages. A total of 14 text messages were developed to be sent during the third trimester of pregnancy (weeks 28, 30, 32, 34, 36, 38, 39, and 40), and weekly for the first six weeks after delivery. Messages were personalized by having an option to insert the recipient's name (or their infant's name) and were translated into Kiswahili and Dholuo.

**Table 3:** Intervention text messages.

Gestation Week	Message
28	Hi [name]! Congratulations for visiting clinic this week! Please call or flash 0788100133 if you have questions about your pregnancy. We're here to help you!
30	Hi [name]. We wish you a good and healthy pregnancy. We are here to support you during this journey. If you have questions please call or flash 0788100133
32	Hi [name]! We would like to wish you a good day. Please remember that if you have questions about your pregnancy, you can call or flash 0788100133
34	Good day [name]! Have you visited the mother and child care clinic lately? If not, please feel welcome to visit. Call or flash 0788100133 for questions
36	Greetings [name]! We are here for you if you have any questions about your pregnancy. Please call or flash or send a please call me to 0788100133
38	Hello [name]! Have you planned where you will deliver your baby? Please call or flash 0788100133 if you have questions or want to discuss your options
39	Hi [name], We wish you a healthy pregnancy and safe delivery! If you would like to plan your delivery, call or flash or send please call me to 0788100133
40	Hi [name], We wish you a healthy pregnancy and safe delivery! If you would like to plan your delivery, call or flash or send please call me to 0788100133

Weeks after delivery	
1	Dear [name], congratulations on the birth of baby [babyname]! We treasure you both! If you have questions about baby's health please call or flash 0788100133
2	Dear [name], your baby [boy/girl] will need immunization at 6 weeks of age to prevent childhood diseases and grow healthy and strong. Please call or flash 0788100133 to find out more about baby's clinic schedule. Thank you
3	Hi [name]! How are you & baby [babyname]? We know you're working hard to care for [babyname]. If you have any fever, pain or bleeding, please come to clinic
4	Dear [name], your baby [boy/girl] is now one month old. Congratulations! Please remember to have enough rest and sleep to keep yourself healthy
5	Hi [name]! How are you and baby [babyname]? Your baby needs to be immunized to prevent childhood diseases. Kindly bring baby to clinic next week. See you then!
6	Hi [name]! This week, please bring baby [babyname] to clinic for important immunizations to prevent childhood diseases and make sure [babyname] grows up healthy and strong. You will also be counselled on how to keep your baby [boy/girl] healthy

## Discussion

In this study, we used a multi-stage content development approach to crafting SMS text messages, informed by behavioral theory and prior empirical research. We found that applying this careful and theoretically informed approach resulted in message content that was consistent across different focus groups. We identified certain key themes that we felt would be useful in the design of text messaging content, as they may facilitate women's self-confidence and perceived self-efficacy to attend clinic, reduce or minimize potential fears, and provide “cues to action”. These included maximizing “a personal touch” in messaging, encouragement and validation of the patient, and content that suggested that health care workers were caring and loving toward their patients. Participants held a strong consensus that personalized and caring messages would provide a stimulus to trigger women to take the necessary action of attending clinic. For women who fear knowing the HIV status of their children, such messages could potentially reduce their fears and increase their confidence.

It emerged that reminders about immunizations might help build on women's already clear sense of susceptibility to disease and knowledge of the benefits of treatment. This mix of perceived susceptibility and benefit of immunization can be leveraged to get women back to the clinic, with SMS text messages functioning as “cues to action”. In other cases, findings pointed to stigma as a major barrier to follow-up. Participants unanimously recommended to never use the word “HIV” or other terminology that would suggest HIV testing or infection. The high level of perceived HIV stigma expressed by the participants in our study is consistent with prior research among pregnant women in the Nyanza region [19]; a recent study among women attending antenatal clinics found that 32% anticipated break-up of their relationship, and 45% anticipated losing their friends, if they were to test HIV positive. Women who anticipated negative responses from male partners were especially likely to refuse HIV testing. Our study further suggests that the decision to have infants tested for HIV is intertwined with other issues related to testing and disclosure faced by women, who must often navigate between their fears of HIV/AIDS stigma, the threat of violence from male partners, and their desire to protect their infants from HIV infection. At the same time, many participants felt that involvement of male partners greatly enhances the support that pregnant and postpartum women need with regard to clinic attendance, and that male partners may even take the responsibility of reminding women about clinic visits. Involving male partners could also potentially help to reduce stigma as it could permit a safer space in which marital partners can learn each other's status, and support each other with disclosure and coping with the community around them [19]. In sum, text messaging interventions need to be designed in a way that supports the involvement of men as partners, while being sensitive to the reality that women are at risk of domestic violence.

**Figure 1:**
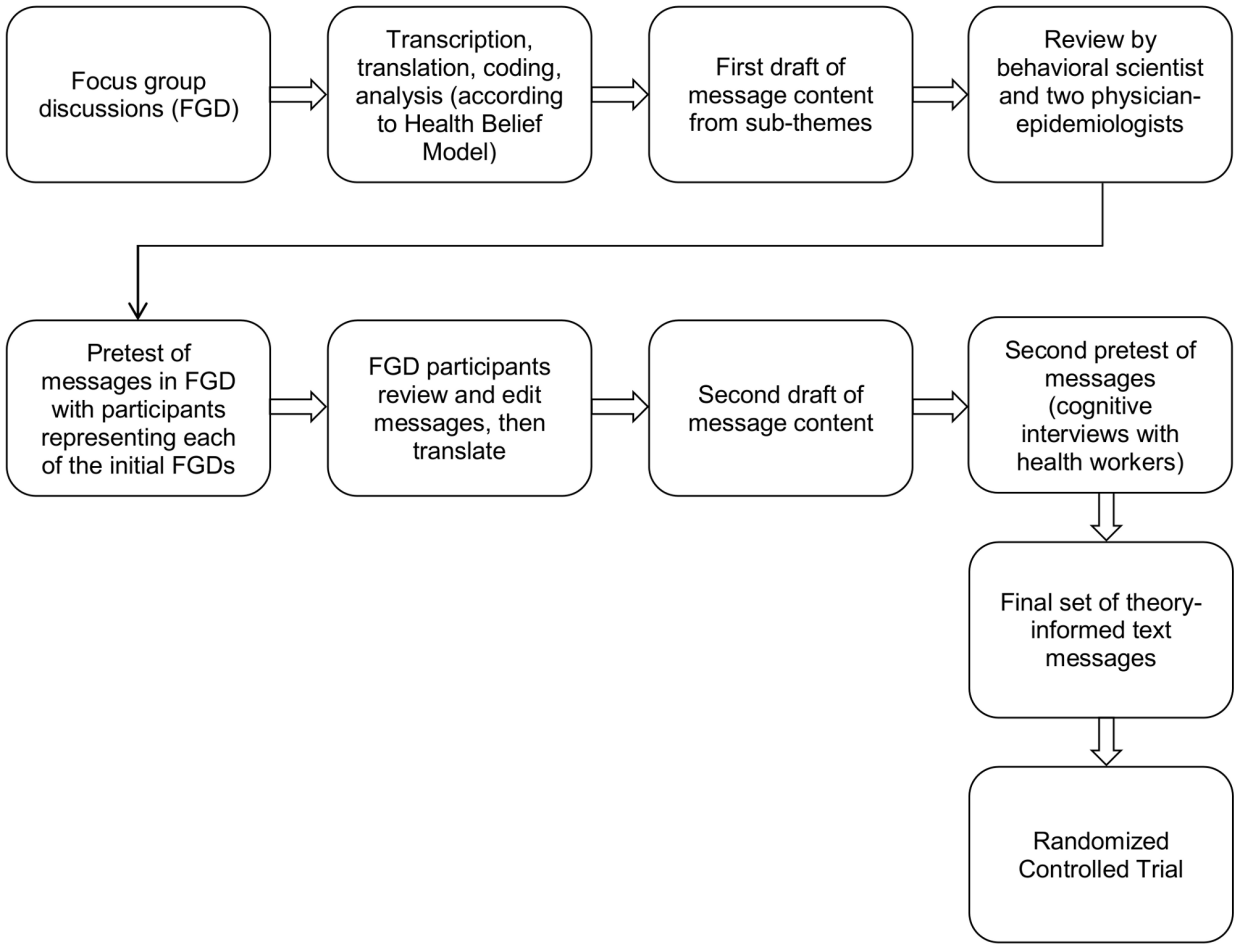
Process of crafting and refining text message content. HBM, Health Belief Model; FGD, Focus Group Discussion.

In the end, our SMS text messaging intervention was designed carefully to minimize women's fears of HIV testing and risks of disclosure – no text messages referred to HIV testing directly. We encouraged women to visit the clinic as scheduled, and provided them a service for texting or calling to ask questions or receive additional information from a clinician. Further, we ensured that text messages were brief and began with a greeting. Messages sent in the post-partum period congratulated mothers and encouraged them to get their children immunized. We also provide a description of the process used to craft these messages (summarized in Figure 1) that would permit replication in other settings.

This study had several limitations. First, given the limited financial resources available, we recruited participants passively and only included women who were present at the clinics. This might have resulted in more informed, motivated women being included. Women who fail to attend clinic might have different perceptions about the use of text messages to improve clinic return and uptake of early infant diagnosis. Similarly, FGD responses may be biased in favor of participants willing to participate in group activities and therefore more likely to have disclosed their status. Second, we did not involve men in any of the patient discussion groups, or have a separate discussion with men. It emerged that male involvement may be a strong determinant of women's ability to make decisions surrounding their health and that of their children. However, our focus group with health workers included male providers who were able to give both professional and personal opinions. Third, participants were all recruited from urban clinics. Women from rural areas also stand to benefit from interventions to improve clinic return, and their input would be useful in crafting messaging content. Finally, although participants generally agreed on the preferred time of day for receiving messages, no consensus emerged regarding the frequency of messages. Other studies have shown that less frequent messages have a stronger association with good clinical outcomes [9]. This informed our final decision to use two-weekly intervals during pregnancy, and weekly intervals after delivery. Our findings might be important for dissemination and implementation practice as relates to mHealth interventions to support HIV programs, providing a model for a multi-stage, theoretically-informed approach to SMS messaging content development. Using theory-based methods could help to answer the “why” when such interventions are successful. Insight from theory-based research could therefore help to understand the effectiveness of mHealth interventions and inform expansion and replication in different regions and settings.

## Conclusions

Text messages may prove useful as a tool to promote and support clinic attendance for pregnant and postpartum women, and to improve the rates of early infant diagnosis. Involving potential message recipients in the design of the intervention by having them craft and refine the actual content of messages, and underpinning the process on sound behavioral theory, resulted in more relevant content for engaging the target audience. Importantly, since our process of crafting these messages was founded on a theoretical framework, it is possible to explain *how* and *why* they can potentially result in behavior change. This also makes it possible for our intervention, and our methods of intervention development, to be adapted for use in other regions and in different contexts. We are evaluating the effect of these messages on improving retention in postpartum HIV care for mother-infant pairs in a randomized trial [28].

## Supporting Information

Table S1 Representative recommendations offered by focus group participants.
(DOCX)

## References

[1] Perez F, Orne-Gliemann J, Mukotekwa T, Miller A, Glenshaw M, et al (2004) Prevention Of Mother To Child Transmission Of Hiv: Evaluation Of A Pilot Programme In A District Hospital In Rural Zimbabwe. Bmj (Clinical Research Ed) 329: 1147. 10.1136/bmj.329.7475.1147 PMC527692 15539670

[2] Unaids (2011) Epidemic Update And Health Sector Progress Towards Universal Access. Global Hiv/Aids Response.

[3] Nassali M, Nakanjako D, Kyabayinze D, Beyeza J, Okoth A, et al (2009) Access To Hiv/Aids Care For Mothers And Children In Sub-Saharan Africa: Adherence To The Postnatal Pmtct Program. Aids Care 21: 1124–1131. 20024771 10.1080/09540120802707467

[4] Manzi M, Zachariah R, Teck R, Buhendwa L, Kazima J, et al (2005) High Acceptability Of Voluntary Counselling And Hiv-Testing But Unacceptable Loss To Follow Up In A Prevention Of Mother-To-Child Hiv Transmission Programme In Rural Malawi: Scaling-Up Requires A Different Way Of Acting. Trop Med Int Health 10: 1242–1250. 16359404 10.1111/j.1365-3156.2005.01526.x

[5] Rawizza H, Meloni S, Oyebode T, Sagay S, Adewole I, et al.. (2012) Evaluation Of Loss To Follow-Up Within The Pmtct Care Cascade In A Large Art Program: Nigeria. 19th Conference On Retroviruses And Opportunistic Infections Paper #1017. Seattle, Washington, Usa.

[6] Odeny Ta, Bailey Rc, Bukusi Ea, Simoni Jm, Tapia Ka, et al (2012) Text Messaging To Improve Attendance At Post-Operative Clinic Visits After Adult Male Circumcision For Hiv Prevention: A Randomized Controlled Trial. Plos One 7: E43832. 22957034 10.1371/journal.pone.0043832 PMC3434192

[7] Elhadj As (2010) Opening Remarks By Unicef Regional Director Elhadj As Sy At The Global Fund Pmtct Meeting. Http//Www.Unicef.Org/Esaro/5440_6380.Html.

[8] Lester Rt, Ritvo P, Mills Ej, Kariri A, Karanja S, et al (2010) Effects Of A Mobile Phone Short Message Service On Antiretroviral Treatment Adherence In Kenya (Weltel Kenya1): A Randomised Trial. Lancet 376: 1838–1845. 21071074 10.1016/S0140-6736(10)61997-6

[9] Pop-Eleches C, Thirumurthy H, Habyarimana Jp, Zivin Jg, Goldstein Mp, et al (2011) Mobile Phone Technologies Improve Adherence To Antiretroviral Treatment In A Resource-Limited Setting: A Randomized Controlled Trial Of Text Message Reminders. Aids (London, England) 25: 825. 10.1097/QAD.0b013e32834380c1 PMC3718389 21252632

[10] Horvath T, Azman H, Kennedy Ge, Rutherford Gw (2012) Mobile Phone Text Messaging For Promoting Adherence To Antiretroviral Therapy In Patients With Hiv Infection. Cochrane Database Of Systematic Reviews (Online) 3: Cd009756. 10.1002/14651858.CD009756 PMC6486190 22419345

[11] Chi Bh, Stringer Js (2010) Mobile Phones To Improve Hiv Treatment Adherence. Lancet 376: 1807. 21071073 10.1016/S0140-6736(10)62046-6

[12] Thirumurthy H, Lester Rt (2012) M-Health For Health Behaviour Change In Resource-Limited Settings: Applications To Hiv Care And Beyond. Bulletin Of The World Health Organization 90: 390. 22589574 10.2471/BLT.11.099317 PMC3341690

[13] Glanz K, Rimer Bk (1997) Theory At A Glance: A Guide For Health Promotion Practice: U.S. Dept. Of Health And Human Services Public Health Service National Institutes Of Health National Cancer Institute.

[14] The Pme (2013) A Reality Checkpoint For Mobile Health: Three Challenges To Overcome. Plos Med 10: E1001395. 23468597 10.1371/journal.pmed.1001395 PMC3582561

[15] Coomes Cm, Lewis Ma, Uhrig Jd, Furberg Rd, Harris Jl, et al (2012) Beyond Reminders: A Conceptual Framework For Using Short Message Service To Promote Prevention And Improve Healthcare Quality And Clinical Outcomes For People Living With Hiv. Aids Care 24: 348–357. 21933036 10.1080/09540121.2011.608421

[16] Riley Wt, Rivera De, Atienza Aa, Nilsen W, Allison Sm, et al (2011) Health Behavior Models In The Age Of Mobile Interventions: Are Our Theories Up To The Task? Transl Behav Med 1: 53–71. 21796270 10.1007/s13142-011-0021-7 PMC3142960

[17] Catania Ja, Kegeles Sm, Coates Tj (1990) Towards An Understanding Of Risk Behavior: An Aids Risk Reduction Model (Arrm). Health Educ Q 17: 53–72. 2318652 10.1177/109019819001700107

[18] (2010) Kenya Demographic And Health Survey 2008-09. Calverton, Maryland: Kenya National Bureau Of Statistics (Knbs) And Icf Macro.

[19] Turan Jm, Bukusi Ea, Onono M, Holzemer Wl, Miller S, et al (2011) Hiv/Aids Stigma And Refusal Of Hiv Testing Among Pregnant Women In Rural Kenya: Results From The Mamas Study. Aids Behav 15: 1111–1120. 20827573 10.1007/s10461-010-9798-5 PMC3127002

[20] Lewis Kulzer J, Penner Ja, Marima R, Oyaro P, Oyanga Ao, et al (2012) Family Model Of Hiv Care And Treatment: A Retrospective Study In Kenya. J Int Aids Soc 15: 8. 22353553 10.1186/1758-2652-15-8 PMC3298805

[21] Rosenstock I, Strecher V, Becker M (1994) The Health Belief Model And Hiv Risk Behavior Change; Diclemente Rj, Peterson Jl, Editors: Springer.

[22] Janz Nk, Becker Mh (1984) The Health Belief Model: A Decade Later. Health Education Quarterly 11: 1. 6392204 10.1177/109019818401100101

[23] Mack N, Woodsong C, Macqueen Km, Guest G, Namely E (2005) Qualitative Research Methods: A Data Collector's Field Guide. Research Triangle Park, North Carolina: Family Health International.

[24] Bandura A, Mcclelland Dc (1977) Social Learning Theory.

[25] Bandura A (1989) Perceived Self-Efficacy In The Exercise Of Control Over Aids Infection. In: Mays Vm, Albee Gw, Schneider Sf, Editors.Primary Prevention Of Aids: Psychological Approaches.London: Sage Publications, Inc. Pp. 128–141.

[26] Rosenstock Im, Strecher Vj, Becker Mh (1988) Social Learning Theory And The Health Belief Model. Health Education Quarterly 15: 175. 3378902 10.1177/109019818801500203

[27] Tourangeau R (1984) Cognitive Sciences And Survey Methods. In: Jabine T, Straf M, Tanur J, Tourangeau R, Editors.Cognitive Aspects Of Survey Methodology: Building A Bridge Between Disciplines: Report Of The Advanced Research Seminar On Cognitive Aspects Of Survey Methodology: National Academies. Pp.73–100.

[28] Odeny T, Bukusi E, Cohen C, Camlin C, Yuhas K, et al.. (2014) Texting Improves Testing: A Randomized Trial Of 2-Way Sms To Increase Postpartum Pmtct Retention And Infant Hiv Testing. Aids: In Press. 10.1097/QAD.0000000000000409 PMC4834137 25313586

