# Supplementary material for: Developing Content for a mHealth Intervention to Promote Postpartum Retention in Prevention of Mother-To-Child HIV Transmission Programs and Early Infant Diagnosis of HIV: A Qualitative Study
Source: PLoS One. 2014 Sep 2;9(9):e106383. doi: 10.1371/journal.pone.0106383 (PMC4152282; doi:10.1371/journal.pone.0106383)
Supplement: Table S1 — Representative recommendations offered by focus group participants. (DOCX) [file pone.0106383.s001.docx]

**Table S1. Representative recommendations offered by focus group participants.**

| **Theme** | **Representative Quote** | **Respondent** |
| --- | --- | --- |
| **Messages should be brief** | “It should be an educative message and should be precise, I mean very short. For informing her of the importance of taking the baby to the clinic.” | 20-year old married HIV-negative woman with no child |
|  | “You know SMS is a brief statement and you cannot make it too long. You just have to look for few words which can comfort someone or sound polite.” | 35-year old married HIV-positive woman with 1 child |
|  | I would prefer ‘Bring the baby to the clinic’. | 23-year old married HIV-negative woman with 2 children |
| **Focus on immunization** | “I think if a message is to be sent it should be like this ‘there is some immunization to be given against some disease, so bring your babies to be immunized to prevent them from that disease.’” | 23-year old married HIV-negative woman with 2 children |
| **Appointment reminders helpful as cues to action** | “Let us assume I am the doctor, I will have records to show that this particular baby is being brought to the clinic for this and that. Therefore when I send a message, it will be tailored to let her know that she is supposed to return the baby to the clinic for vaccination, BCG or polio, something like that. Yes, the date of appointment and what you are coming for.” | 22-year old married HIV-negative woman with 1 child |
|  | “For that message, I will send something to do with, ‘Bring the baby to learn his status so that we help him’” | 22-year old single HIV-negative woman with 1 child |
|  | “The message should be framed as ‘bring the baby to the clinic’ then when she comes to the clinic, she will be told about the rest like the plan for VCT.” | 20-year old married HIV-negative woman with 1 child |
|  | “If you send me a message telling me that my appointment date has reached, so I should bring my baby to the clinic.” | 32-year old married HIV-negative woman with 1 child |
|  | “She can be reminded according to the next appointment date to take the baby to the clinic for vaccination. On this she may be sent an SMS and when at the hospital, the mothers can be gathered together just the way you did this to us today then the health providers who are qualified in counselling may approach her.” | 24-year old married HIV-negative woman with 2 children |
| **Convey warmth and ‘personal touch’** | “If in that message they address me by name I will know they recognize me. You know if one calls you by name you know at least they recognize you and it is not like they sent the SMS for granted. So that would mean they know me and want me to go. In the first place, that would motivate me to go.” | 27-year old married HIV-negative woman with no child |
|  | “First of all I would consider how C2 would perceive my message of I have to be aware how to best communicate with her so that when I type this message, I may start by calling her the beautiful one and asking her if she remembers the date she is supposed to go to the clinic.” | 36-year old married HIV-positive woman with 4 children |
|  | “All of us, like human beings, we want to be loved. Everybody would wish to be loved. Therefore you may start with some appealing message to catch her attention. Meaning, the first word you use must sound loving, depending on how close you are with her or showing that kind of love to her. I want to say so.” | 35-year old married HIV-positive woman with 1 child |
|  | “I wanted to say that if this is a mother who has delivered and wants to return the baby to the clinic after six weeks for the test, I can encourage her to try her best… So you just have to encourage her. You can send her an SMS for encouragement.” | 36-year old married HIV-positive woman with 4 children |
| **Mothers need congratulations** | “First of all I may really want someone to congratulate me because this journey is really long; to reach the destination is no joke. After you’ve congratulated me I think I’ll feel happy coz we all need to be congratulated for the success.” | 27-year old married HIV-negative woman with no child |
| **Begin with polite greetings** | “First is good morning Mrs. So and So. Congratulations. The day, tomorrow is your day for this and this, please come for this and this, the benefits, then you write the benefits.” | 26-year old married HIV-negative woman with no child |
|  | “…you will start good morning, tomorrow is your day for…” | 24-year old married HIV-negative woman with no child |
|  | “Let me say you start with the greetings and then you request kindly come.” | 28-year old married HIV-negative woman with 2 children |
|  | “…you greet her first and ask how she is fairing on together with her family and children.” | 30-year old married HIV-positive woman with no child |
|  | “Yes, to remind her to come to the clinic, they need to say greetings, maybe how she has woken up, how she is doing, if she has delivered, if she has come to the clinic. If she has not come to the clinic, then welcome her to the clinic. I mean things like that, just to persuade the mother.” | 22-year old married HIV-negative woman with 3 children |
| **Polite greetings** | “You may start by requesting her so that you get along well because if you may shock her if you put it in a bad way. You can just remind her of when she is supposed to go for her next appointment.” | 28-year old married HIV-positive woman with 1 child |
|  | “You may use words like please, kindly and others like these. It could be one word that can generalize the others and after putting it that way, she will say for sure this person has talked to me politely and humbly.” | 35-year old married HIV-positive woman with 1 child |
| **Avoid discussion of HIV or VCT*** | “But you should not send one like this: ‘Please come, bring the baby for HIV test’, no.” | 22-year old married HIV-negative woman with 3 children |
|  | “You know you can surely shock someone when you tell her about VCT. If you tell her to bring the baby for VCT and maybe she is also infected, she will get shocked and conclude that the baby has contracted the virus. You know the term VCT is a very strong word and if someone sends to you a message with such a word, you will fear.” | 22-year old single HIV-negative woman with 1 child |

* VCT, voluntary counselling and testing
